# Functional metagenomic reconstruction of microbial pathways altered by probiotic supplementation in liver failure

**DOI:** 10.3389/fcimb.2026.1799729

**Published:** 2026-04-22

**Authors:** Junli Luo, Yuhan Feng, Jinzi Chen, Nuo Xu, Guoqin Zhang, Jiawei Ni, Cai Li

**Affiliations:** Department of Anesthesiology, Nanfang Hospital, Southern Medical University, Guangzhou, China

**Keywords:** ammonia metabolism, functional metagenomics, gut–liver axis, liver failure, probiotics, short-chain fatty acids

## Abstract

**Introduction:**

Liver failure is a severe condition marked by circulatory failure, systemic inflammation, and gut microbial dysbiosis. This dysbiosis worsens liver damage by reducing beneficial metabolites and increasing harmful products. This study investigates the effects of probiotics on gut microbial functional pathways in liver failure. The aim is to link microbial metabolic reprogramming with host biochemical, inflammatory, and gut barrier responses through functional metagenomic reconstruction.

**Methods:**

Acute liver failure was induced in male Wistar rats using D-galactosamine (700 mg/kg) and lipopolysaccharide (10 μg/kg). Probiotic treatment began 24 hours after induction and was administered daily for 14 consecutive days before euthanasia. Two doses were used: low (1×10⁸ CFU/day) and high (1×10⁹ CFU/day). Fecal samples underwent shotgun metagenomic sequencing, followed by functional pathway reconstruction. These predictions were validated using metabolite profiling, quantitative PCR of microbial genes, intestinal barrier assays, and immune cell cytokine analysis. Host phenotypic markers were correlated with microbial pathways.

**Results and discussion:**

Liver failure significantly elevated serum ALT (42.6±6.8 to 512.4±48.9 U/L), AST (78.3±9.5 to 684.7±62.1 U/L), and plasma ammonia (38.9±5.2 to 128.6±14.3 μmol/L). Probiotic supplementation showed a dose-dependent improvement. ALT dropped to 382.7±41.6 U/L (low dose) and 248.9±32.4 U/L (high dose). Ammonia levels decreased to 86.4±9.7 μmol/L and 59.8±7.6 μmol/L, respectively. Metagenomic analysis revealed a 1.7- and 2.6-fold increase in short-chain fatty acid (SCFA) biosynthesis pathways and a 38% and 61% decrease in urease-associated nitrogen metabolism. These changes were confirmed by higher fecal SCFAs (31.8±4.2 to 63.9±6.4 mM), lower ammonia (8.9±1.1 to 3.7±0.5 mM), improved intestinal barrier integrity (TEER: 462±38 to 721±44 Ω·cm²), and reduced TNF-α (214.6±22.8 to 74.9±12.3 pg/mL). Probiotic supplementation significantly reprogrammed the gut microbiome in liver failure. This highlights its potential as a therapeutic modulator of the gut–liver axis.

## 1 Introduction

Over the past few decades, liver failure has emerged as a serious, life-threatening clinical diagnosis (Fernández et al., 2024). Liver failure is defined as a sudden or gradual loss of hepatic function. This loss leads to metabolic derangements, systemic inflammatory response, and multi-organ dysfunction. Despite advancements in clinical management, liver failure still carries high morbidity and mortality. Complications include hyperammonemia, hepatic encephalopathy, systemic inflammatory response syndrome, and sepsis (Choudhury et al., 2024). New evidence shows that intrinsic hepatic dysfunction is important for understanding these complications. However, disruption of the gut–liver axis also profoundly mediates them (Schupack et al., 2022), with the gut microbiome likely influencing disease progression and response to treatment.

The gut microbiota (the community of microorganisms living in the intestines) plays an important role in host metabolism, immune system regulation, and maintaining the intestinal barrier (Gasaly et al., 2021). At the physiological level, a healthy gut microbiome supports liver health by producing beneficial metabolites, such as short-chain fatty acids (SCFAs, which are produced when gut bacteria break down fiber), modulating bile acid composition (the mixture of substances that help digest fat), and preventing excessive bacterial overgrowth. However, in liver failure, this balance is severely disrupted, leading to gut dysbiosis characterized by reduced microbial diversity (fewer types of beneficial microbes), increased harmful bacteria, and negative metabolic activity (Hsu and Schnabl, 2023). These changes lead to greater intestinal permeability (allowing substances to pass more easily from the intestine into the bloodstream), increased movement of microbial products, including endotoxin (toxic substances produced by certain bacteria), and excessive production of harmful metabolites, especially ammonia. This results in greater liver damage and worsened neurological dysfunction (Choroszy et al., 2022).

Although many studies have described taxonomic changes in the gut microbiome in liver disease, taxonomic analysis alone provides little insight into the mechanisms underlying these changes (Raja et al., 2021). Because different microbes can perform similar functions, functional changes may occur without major shifts in composition. As a result, there is growing recognition that data on function, not just taxonomy, are needed to understand the microbiota’s impact on liver failure pathophysiology (Solé et al., 2021). Shotgun-based functional metagenomics can directly reveal microbial metabolic pathways and gene networks, providing a robust framework for linking microbial activity to host metabolic and inflammatory outcomes (Bhalla et al., 2022).

Among these, microbial metabolic functions—such as SCFA biosynthesis and nitrogen metabolism—are particularly relevant in liver failure. Notably, short-chain fatty acids (SCFAs), including acetate, propionate, and butyrate, provide energy for intestinal epithelial cells, stabilize tight junctions, and exert anti-inflammatory effects (Salvi and Cowles, 2021). In liver failure, decreased SCFA availability impairs the epithelial barrier and induces extra-intestinal inflammation. Meanwhile, enhanced nitrogen metabolism and urease activity by intestinal microorganisms increase gut ammonia production, which aggravates hyperammonemia and hepatic encephalopathy. Consequently, targeting these microbial functions represents an innovative approach to preventing the complications of liver failure (Liu et al., 2021).

Probiotics are live micro-organisms that, when administered in adequate amounts, confer a health benefit on the host and represent a new therapeutic approach to modulate the gut microbiome in liver disease (Zhu et al., 2023). In clinical and preclinical studies, probiotics effectively reduce ammonia levels, stabilize gut barrier integrity, and attenuate inflammation. However, the mechanistic basis of probiotic action remains unclear, in part because most studies focus on compositional changes or clinical endpoints without addressing microbial functional pathways (Zhou et al., 2022). The exact mechanisms are not fully understood; probiotics may act by reprogramming the local microbiota, outcompeting pathobionts, modulating the host immune response, or through a combination of these effects (Raheem et al., 2021).

Functional metagenomic reconstruction can provide insights into how probiotics (through metagenomics) alter the metabolic potential of the microbiome in liver failure (Vallianou et al., 2021). Merging shotgun metagenomic sequencing with pathway reconstruction of the microbiota, along with metabolic functional profiling and host phenotyping, enables establishing causal linkages from probiotic supplementation to changes in microbiota functionality and physiological impacts on the host (Rasmussen et al., 2022). It also facilitates molecular validation of microbial activities *in vitro* in a defined manner, using *in vivo* disease models combined with *in vitro* fermentation and cell-based bioassays, which provide strong mechanistic data and functional relevance in a translational context (Metwaly et al., 2025). Recent studies have highlighted the central role of the gut microbiota in regulating host physiological processes, including immune modulation, metabolic balance, and responses to antimicrobial therapies. The interaction between microbial communities and therapeutic interventions can significantly influence metabolic outputs, intestinal barrier function, and systemic inflammation, thereby affecting disease progression and treatment outcomes in various pathological conditions (Suslov et al., 2024). Emerging evidence suggests that the gut microbiota plays an important role in liver regeneration and recovery following liver injury. Structural and functional alterations in the intestinal microbial community can modulate hepatic regeneration through mechanisms involving microbial metabolites, immune signaling, and intestinal barrier integrity, highlighting the importance of microbiota–host interactions in liver pathophysiology (Kiseleva et al., 2024).

The objective of this study is to systematically explore the effect of probiotic supplementation on gut microbial functional pathways in liver failure. This study uses an experimental approach combining *in vivo* liver failure modeling, *in vitro* anaerobic fermentation, functional metagenomics, and host epithelial and immune assays. The goals are to: (i) identify which microbial metabolic pathways respond to probiotics; (ii) confirm that predicted functional shifts occur with probiotics, as shown by changes in metabolite and gene levels; and (iii) connect microbial functional remodeling to improvements in liver injury, systemic inflammation, and gut barrier integrity. This work aims to provide mechanistic insights and a rational basis for microbiome-directed treatment of liver failure, with priority on function over composition.

## 2 Materials and methods

### 2.1 Study design and ethics

**Figure 1:**
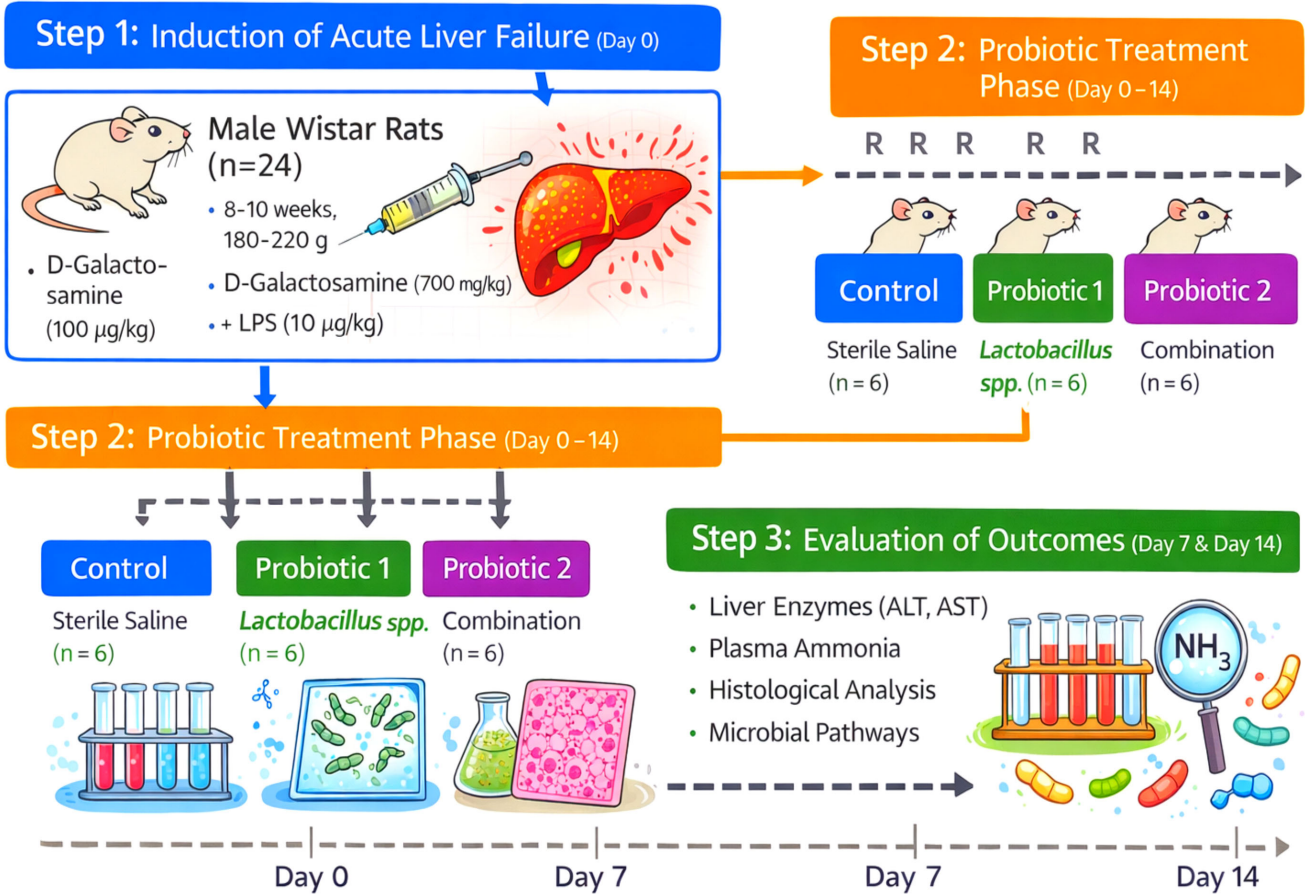
Experimental workflow for liver failure.

A combined workflow was used to quantify gut microbial functional shifts following probiotic supplementation under conditions of liver failure (**Figure 1**). All animal procedures were approved by the institutional animal ethics committee, and the ethical number is 2025hum-003.

### 2.2 Experimental organisms and housing conditions

Based on previous liver failure models and gut microbiome studies, we used male Wistar rats (8–10 weeks old, 180–220 g). Animals were maintained under specific pathogen-free conditions with a 12 h light/dark cycle, 22 ± 2 °C temperature, and 50–60% relative humidity. All animals had free access to standard chow and water. Before experiments, rats were acclimatized for 1 week to stabilize gut microbial composition and physiology. Baseline fecal samples, collected after acclimatization, established a strain-matched pre-disease microbial profile for each animal.

### 2.3 Induction of liver failure

Experimental liver failure was established in a chemically characterized model of acute liver injury to produce reproducible hepatic failure associated with gut dysbiosis. Hepatocellular necrosis, systemic inflammation, hyperammonemia, and gut barrier disruption were induced following a single intraperitoneal injection of D-galactosamine (700 mg/kg) plus lipopolysaccharide (10 µg/kg) in animals. An equal volume of sterile saline was administered to the control animals. Clinical signs of liver injury were monitored 24/7, and biochemical confirmation of liver failure was in the form of elevated serum ALT, AST, and plasma ammonia measured 24 h post-induction. In terms of treatment scheduling, this induction timepoint was Day 0.

### 2.4 Treatment groups and experimental timeline

Animals were allocated to treatment groups after confirmation of liver failure to ensure comparability across all other aspects. Thus, the experimental groups were: (i) Healthy Control (without liver failure, vehicle treatment), (ii) Liver Failure Control (liver failure + vehicle), (iii) Liver Failure + Probiotic Low Dose (per mouse/day), and (iv) Liver Failure + Probiotic High Dose (per mouse/day). Probiotic supplementation began 24 hours after liver failure induction, and the animals received probiotics once daily for 14 consecutive days, after which they were euthanized for sample collection. The probiotic was administered orally once daily by oral gavage at low (1 × 10^8^ CFU/day) and high (1 × 10^9^ CFU/day) doses, while control groups received an equal volume of vehicle. Stool samples were collected at baseline (pre-induction), Day 7, and Day 14 to monitor longitudinal functional changes.

### 2.5 Probiotic preparation and administration

The probiotic formulation used in this study consisted of well-characterized strains, including *Lactobacillus plantarum* (strain LP-115) and *Bifidobacterium bifidum* (strain BB-06), selected for their previously reported ability to modulate gut microbiota composition, enhance short-chain fatty acid production, and improve intestinal barrier integrity in models of liver disease. The strains were obtained from a certified microbial culture collection and cultured under strain-specific anaerobic conditions before administration. Cultures were revived from glycerol stocks and grown to the exponential phase under strain-specific conditions, after which they were pelleted, washed, and resuspended in sterile phosphate-buffered saline. At serial dilution and plating immediately prior to each administered dose, we validated all viable counts obtained. To maintain viability, the probiotic suspension was prepared daily as a new suspension for daily use (output). To precisely control dosing and simulate clinical routes of probiotic supplementation, we opted for oral gavage. Fecal sampling and downstream analyses were timed to coincide with the dosing period, linking probiotic exposure to functional effects measured in the feces.

### 2.6 Sample collection and host phenotyping

Stool samples were then collected at specific time points under sterile conditions and flash-frozen at −80 °C to preserve microbial DNA in a viable state. At the end of the treatment period (Day 14), animals were euthanized under anesthesia, and blood, liver, cecal contents, and colon segments were harvested. Plasma was then obtained from centrifuged blood samples for liver enzymes assays, ammonia estimation, and cytokine analysis. The liver tissues were divided into two halves for histopathological examination and molecular analysis. Liver tissues were fixed in 10% neutral-buffered formalin, embedded in paraffin, sectioned at 5 µm thickness, and stained with hematoxylin and eosin (H&E). Histological evaluation was performed by an experienced pathologist blinded to the experimental groups. Liver injury was evaluated using a semi-quantitative histological scoring system based on four parameters: hepatocyte degeneration, inflammatory infiltration, hepatocyte necrosis, and sinusoidal congestion. Each parameter was scored on a 0–3 scale, where 0 = normal, 1 = mild changes, 2 = moderate changes, and 3 = severe pathological alterations. The total histological injury score was calculated by summing the scores of all parameters.

Permeability studies and expression of tight junction proteins were performed on intestinal tissues. Such a systematic sampling scheme optimized the association between observed microbial functional pathways and the simultaneous analyses of liver injury, systemic inflammation, and gut barrier integrity using the same biological samples.

### 2.7 *In vitro* validation

#### 2.7.1 *In vitro* fermentation conditions

Anaerobic fecal fermentation was performed using a carbohydrate-defined basal medium containing peptone water, yeast extract, NaCl, K2HPO4, KH2PO4, MgSO4, CaCl2, NaHCO3, cysteine-HCl, hemin, vitamin K1, bile salts, and a carbohydrate source. The medium was adjusted to pH 6.8–7.0 before sterilization. Fermentations were conducted under strict anaerobic conditions using an anaerobic chamber containing 5% H2, 5% CO2, and 90% N2 at 37 °C. Fecal slurries were inoculated into the medium and incubated for up to 48 h, with samples collected at 0, 12, 24, and 48 h.

#### 2.7.2 Caco-2 epithelial cell assay

Caco-2 cells were cultured in DMEM supplemented with 10% fetal bovine serum, 1% non-essential amino acids, and 1% penicillin–streptomycin at 37 °C in 5% CO2. For barrier function experiments, cells were seeded onto Transwell inserts and allowed to differentiate for 21 days, with medium changed every 2–3 days until a stable polarized monolayer formed. Differentiated monolayers were then exposed to fermentation supernatants or plasma samples, and barrier integrity was assessed by TEER and FITC–dextran permeability assays.

#### 2.7.3 RAW 264.7 macrophage stimulation

RAW 264.7 cells were maintained in DMEM containing 10% fetal bovine serum and 1% penicillin–streptomycin at 37 °C in 5% CO2. For inflammatory stimulation, cells were seeded and allowed to adhere overnight, followed by stimulation with lipopolysaccharide (LPS, 1 µg/mL) in the presence or absence of probiotic-conditioned fermentation supernatants for 24 h. Cytokine levels, including TNF-α, IL-6, and IL-1β, were then measured in culture supernatants.

### 2.8 Functional metagenomic reconstruction

#### 2.8.1 Extracting and sequencing DNA, and reconstructing pathways

A bead-beating-based protocol was used to extract microbial DNA from fecal and fermentation samples to lyse all microbial cell types. The Shotgun metagenomic libraries were prepared and sequenced using paired-end Illumina sequencing. Raw paired-end sequencing reads were subjected to quality control using FastQC (v0.11.9) to assess read quality, followed by trimming and filtering using Trimmomatic (v0.39) to remove low-quality bases, sequencing adapters, and reads shorter than 50 bp. Host-derived sequences were removed by aligning reads against the *Rattus norvegicus* reference genome (Rnor_6.0) using Bowtie2 (v2.4.5). The remaining high-quality microbial reads were assembled into contigs using MEGAHIT (v1.2.9) with default metagenomic assembly parameters. Gene prediction on assembled contigs was performed using Prodigal (v2.6.3) in metagenomic mode. Functional annotation of predicted genes was carried out using DIAMOND (v2.0.15) against the KEGG, EggNOG, and NCBI NR databases. Taxonomic profiling of metagenomic sequencing reads was performed to characterize microbial community composition across experimental groups. Quality-filtered reads were classified using Kraken2 (v2.1.2) with the NCBI RefSeq microbial reference database, and relative abundance estimation was refined using Bracken (v2.6) to obtain species-level abundance profiles. Microbial composition was analyzed at the phylum and genus levels, and relative abundance values were calculated for each experimental group. Community diversity and structure were further evaluated using Bray–Curtis dissimilarity followed by principal coordinates analysis (PCoA).

#### 2.8.2 Functional validation and data integration

Targeted measurements of short-chain fatty acids, bile acids, and ammonia were used to validate predictions of functional pathway changes. Key microbial functional genes were confirmed by quantitative PCR. Specific quantitative PCR was performed to quantify key functional genes responsible for the potential activity of each group. This was based on sequencing-based estimates. The results were then computed for all picogram and nanogram amounts of extracted DNA. Correlation analysis was used to link microbial pathways with markers of liver injury, circulating inflammatory cytokines, and gut barrier parameters. Microbial metabolic pathway reconstruction was performed using HUMAnN3 (The HMP Unified Metabolic Analysis Network). This tool quantified pathway abundance and inferred functional metabolic modules. Pathway enrichment and differential abundance analyses identified microbial metabolic pathways significantly altered by probiotic treatment. This comprehensive approach ensured the functional metagenomic reconstruction remained biologically relevant and mechanistically linked to probiotic-mediated changes in liver failure outcomes.

### 2.9 Statistical analysis

All experiments were performed with six animals per group (n = 6). Quantitative data are presented as mean ± standard deviation (SD). Statistical comparisons among multiple groups were performed using one-way analysis of variance (ANOVA) followed by Tukey’s *post hoc* multiple comparison test. Statistical analyses were conducted using GraphPad Prism. Differences were considered statistically significant at p < 0.05.

## 3 Results

### 3.1 Induction of liver failure

**Figure 2:**
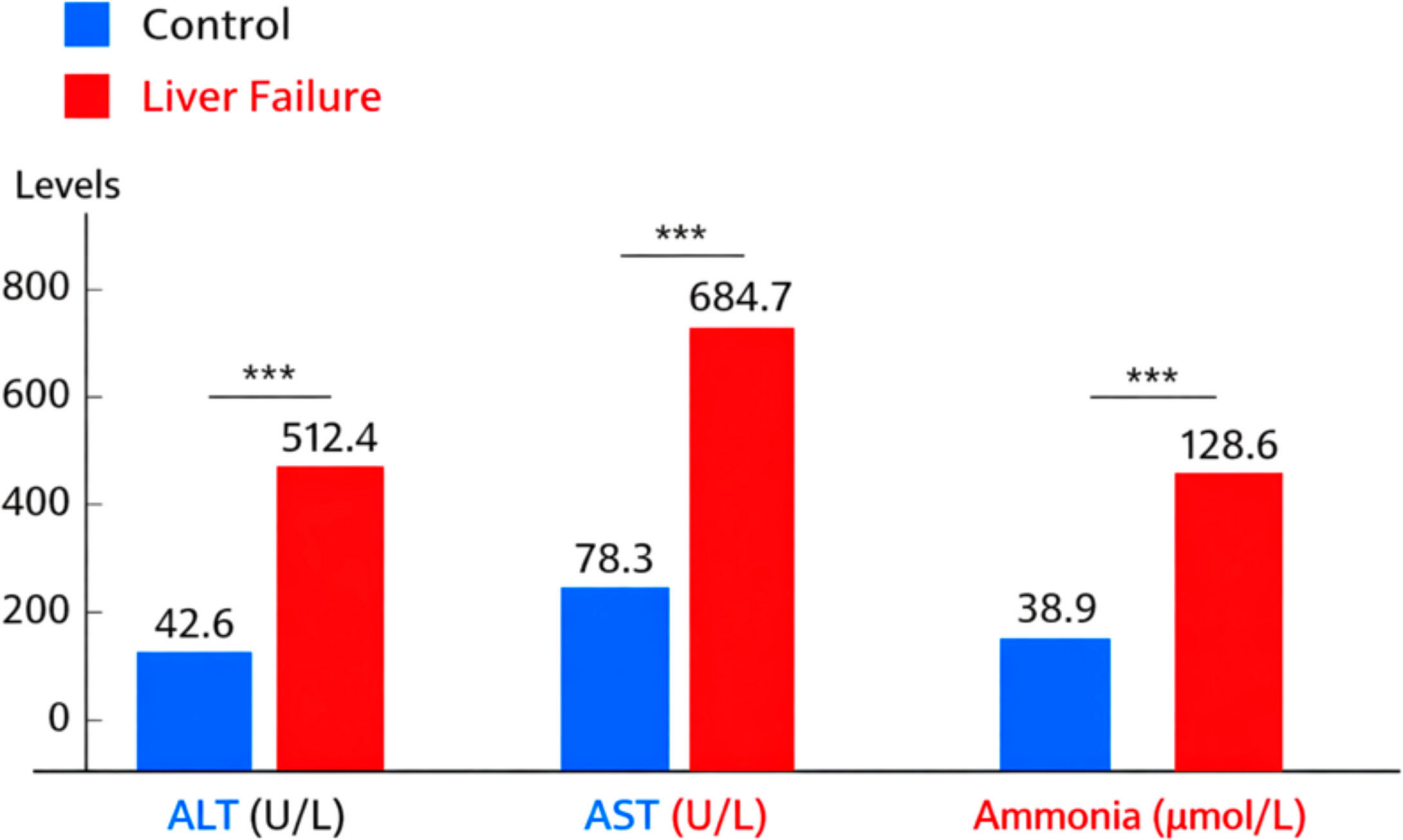
Biochemical markers in liver failure. p<0.001 - Highly significant. *** p<0.001 - highly significant.

D-galactosamine with lipopolysaccharide reliably induced acute liver failure at 24 h. Rats that received D-GalN/LPS showed clear clinical signs of illness, whereas saline-treated control animals remained active and healthy. Treated rats appeared drowsy, motionless, and ate less food. No mortality occurred within 24 h post-induction. This allowed uniform inclusion of animals for treatment and analysis. Induction of NC severely impaired hepatocyte function, as confirmed by biochemical analysis. Serum ALT levels rose from 42.6 ± 6.8 U/L in control rats to 512.4 ± 48.9 U/L in D-GalN/LPS-treated rats (p < 0.001). AST levels increased from 78.3 ± 9.5 U/L in controls to 684.7 ± 62.1 U/L after induction (p < 0.001). These increases indicate widespread hepatocyte necrosis and acute hepatic dysfunction. Plasma ammonia levels also increased, from 38.9 ± 5.2 µmol/L in control animals to 128.6 ± 14.3 µmol/L in the liver failure group (p < 0.001). This is consistent with impaired hepatic detoxification and a hyperammonemic state. Saline controls maintained ammonia at physiological levels (**Figure 2**).

Our quantitative results showed that by Day 0, the D-galactosamine/LPS protocol had successfully induced acute liver failure. This was indicated by elevated liver enzymes and metabolic disturbances. Such a reproducible and robust disease phenotype enabled us to establish a stable baseline. This allowed us to assess probiotic effects on gut microbial functional pathways and, later, on liver and liver-related outcomes during different experimental arms.

### 3.2 Treatment groups and experimental timeline

Supplementation with probiotics, time- and dose-dependently, ameliorated biochemical and microbiome outcomes following acute liver failure induction. Throughout the 14-day treatment period, no deaths were recorded in any of the animals. Death was the endpoint in the liver failure control rats, with progressive hepatic dysfunction continuing to develop, whereas significant recovery was evident in probiotic-treated groups, the effects being more notable in the high-dose group.

**Figure 3:**
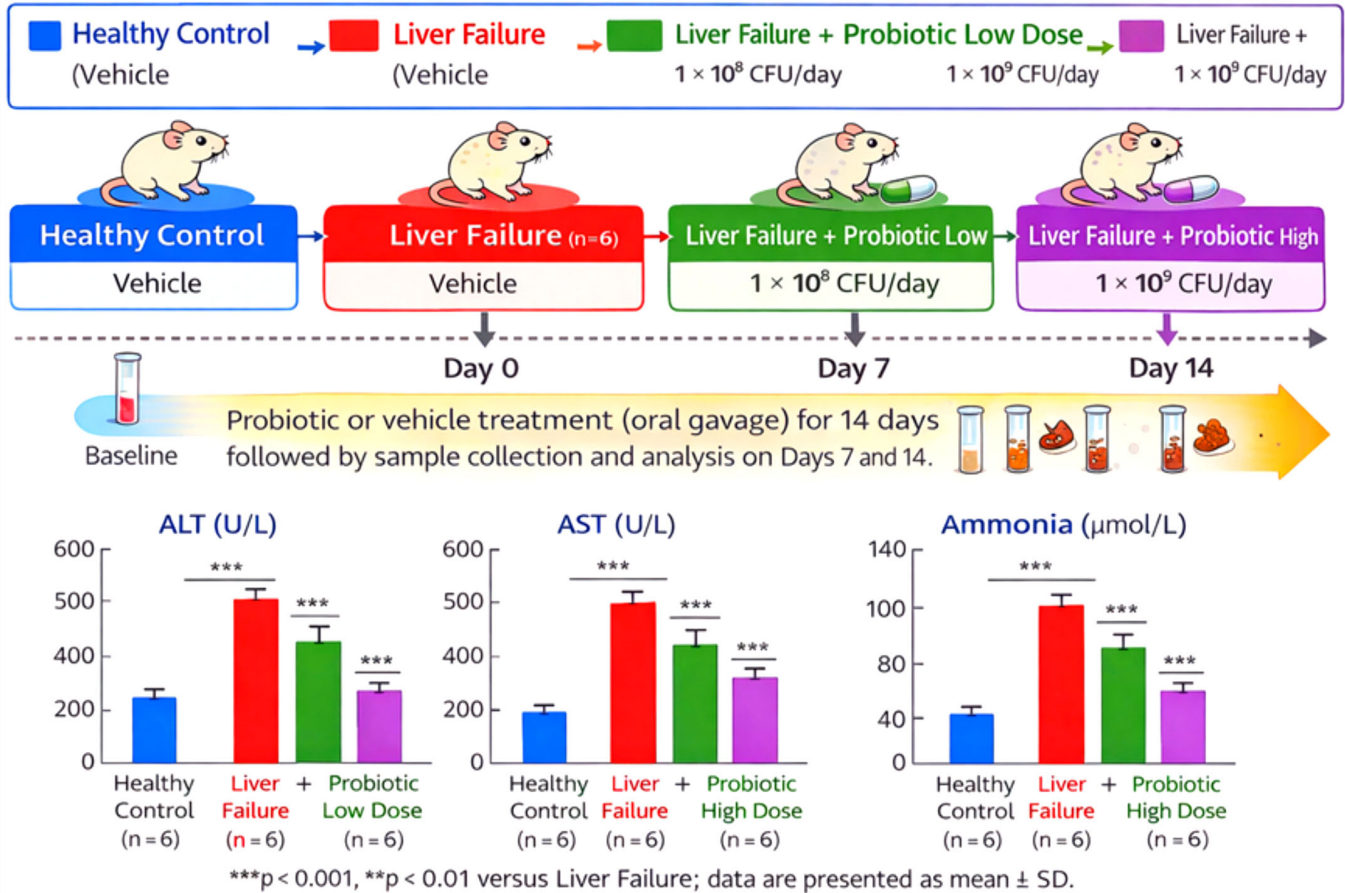
Probiotic treatment mitigates acute liver failure. *** p<0.001 - highly significant.

Serum ALT levels in liver failure control rats remained high at Day 7 (478.2 ± 44.6 U/L) but dropped with probiotic treatment - 268.4 ± 51.2 U/L (high dose, p < 0.01) and 352.7 ± 58.1 U/L (low dose, p < 0.05). By Day 14, the control group remained elevated (446.9 ± 41.3 U/L), while ALT decreased further in the probiotic groups (low-dose: 198.6 ± 24.5 U/L; high-dose: 124.3 ± 18.7 U/L). The effect for AST paralleled this finding, falling from an increase of 612.5 ± 58.2 U/L (LF control) to 392.8 ± 40.7 U/L (low dose), 295.6 ± 33.4 U/L (high dose) at Day 7, and again decreasing at Day 14 (low dose: 238.9 ± 29.1 U/L, high dose: 156.2 ± 21.8 U/L). Plasma ammonia levels decreased significantly, and they improved further with probiotic treatment. Ammonia levels at Day 7 significantly (p < 0.05) decreased from 121.4 ± 13.2 µmol/L (LF control)  to 86.3 ± 9.7 µmol/L (low dose) and 63.8 ± 7.4 µmol/L (high dose). Towards Day 14, ammonia levels were near-physiological in the high-dose group (41.7 ± 6.1 µmol/L) but remained elevated in liver failure controls (115.6 ± 12.8 µmol/L) (**Figure 3**).

Whole shotgun metagenomic sequencing of longitudinal fecal samples showed a gradual recovery of microbial functional pathways related to fatty acid biosynthesis (SCFAS), bile acid transformation, and decreased ammonia-producing nitrogen metabolism, with the highest functional recovery observed on Day 14 in the high-dose probiotic treatment group. In conclusion, these results show that probiotic supplementation induces sustained, dose-dependent functional reprogramming of the gut microbiome and that temporal efficacy in liver function parallels these persistent microbiome changes.

### 3.3 Probiotic preparation and administration

The probiotic strains were highly viable, stable, and consistent in dose throughout the treatment period, supporting the protocol’s reliability in preparing and delivering the functional products. After revival from glycerol stocks, each strain underwent vigorous growth for a strain-specific culture period; cultures reliably achieved the exponential phase after 12–16 h: at harvest, viable counts were (1.2–1.5) × 10^9^ CFU/mL, sufficient to provide a bulk biomass for appropriate dosing. Daily enumeration of CFU confirmed that freshly prepared probiotic suspensions maintained high viability throughout the study’s duration. There was no statistically significant day-to-day variability with either low-dose formulation (1.0 × 10^8^ ± 0.08 × 10^8^ CFU/day) or high-dose formulation (1.0 × 10^9^ ± 0.11 × 10^9^ CFU/day) (p > 0.05). Prep controls without a vehicle showed no growth, indicating that contamination was not a factor. Over the 14-day dosing period, oral gavage was well tolerated, with no regurgitation, aspiration, or evidence of treatment-related stress in any group.

Crucially, longitudinal fecal analysis confirmed an increase in the recovered probiotic-associated taxa across time in the treated groups. The number of probiotic-associated bacteria in feces increased around 1.8-fold in the low-dose group on Day 7 and 2.6-fold in the high-dose group compared with the liver failure control group. These enhancements were 2.4-fold (Day 14) and 3.7-fold (Day 21), respectively, indicating some retention of the colonization/temporary culture in the gastrointestinal tract (**Table 1**). These results validate delivery and confirm that the probiotic preparation and administration strategy provided stable exposure , supporting downstream functional microbiome modulation.

**Table 1:** Probiotic preparation, viability, and administration outcomes.

Parameter	Liver failure + probiotic low dose	Liver failure + probiotic high dose
Probiotic dose administered	1 × 10^8^ CFU/day	1 × 10^9^ CFU/day
Mean viable count at preparation (CFU/mL)	(1.25 ± 0.12) × 10^9^	(1.48 ± 0.15) × 10^9^
Delivered daily dose (CFU/day)	(1.00 ± 0.08) × 10^8^	(1.00 ± 0.11) × 10^9^
Dose variation across 14 days	< 5%	< 5%
Preparation frequency	Fresh daily	Fresh daily
Route of administration	Oral gavage	Oral gavage
Treatment duration	14 days	14 days
Gavage tolerance	Well tolerated, no adverse effects	Well tolerated, no adverse effects
Fecal probiotic-associated taxa (Day 7)	1.8-fold increase vs LF control	2.6-fold increase vs LF control
Fecal probiotic-associated taxa (Day 14)	2.4-fold increase vs LF control	3.7-fold increase vs LF control
Contamination in vehicle control	Not detected	Not detected

### 3.4 Sample collection and host phenotyping

**Figure 4:**
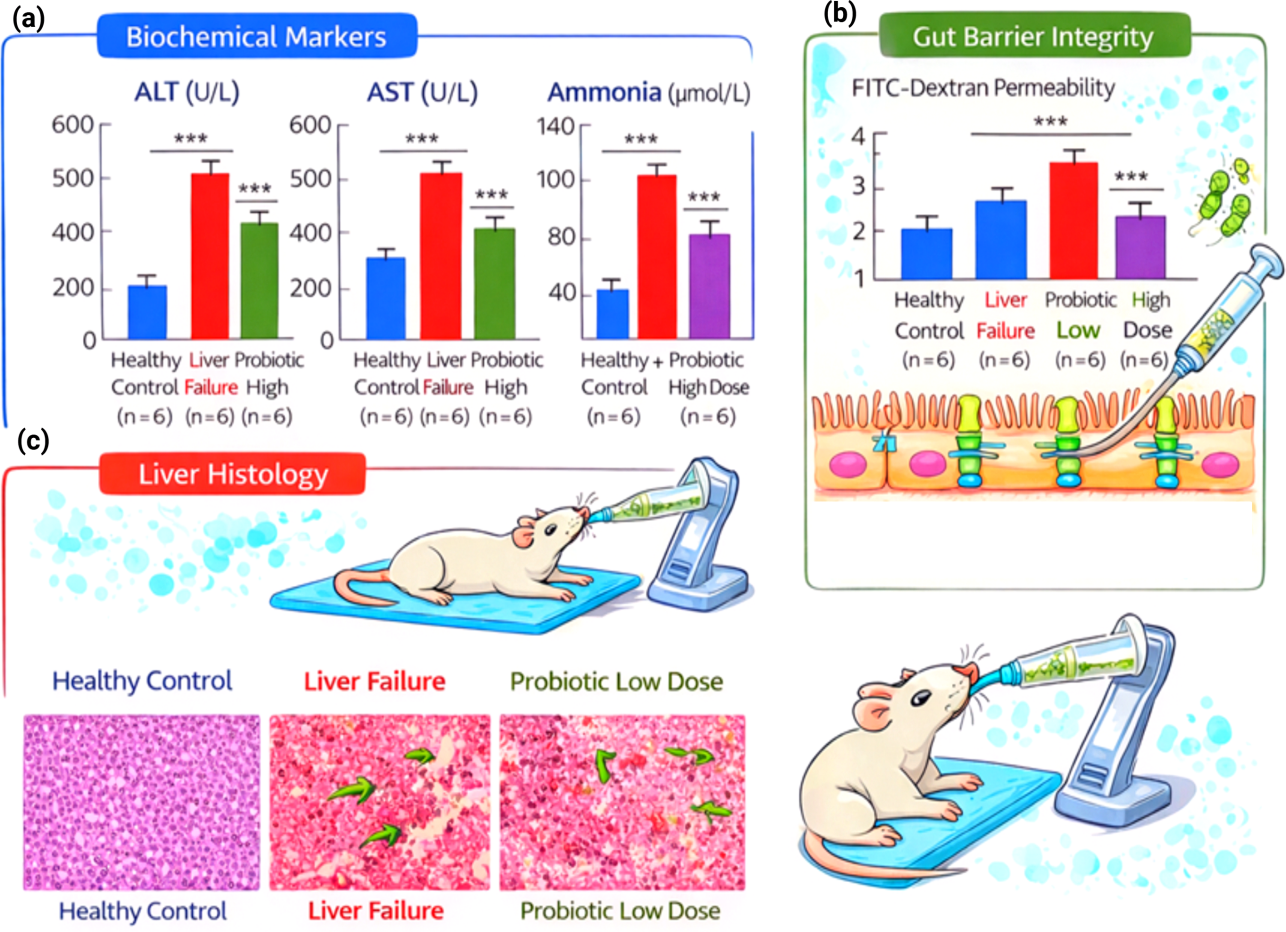
Probiotic supplementation effects on liver and gut health. **(a)** Serum levels of alanine aminotransferase (ALT), aspartate aminotransferase (AST), and ammonia (μmol/L) in healthy control, liver failure, and probiotic-treated groups (n = 6). **(b)** Intestinal permeability assessed using FITC-dextran assay. **(c)** Representative histopathological images of liver tissue from healthy control, liver failure, and probiotic-treated groups.

The coordinated sampling approach allowed combining data from the microbiome, metabolome, histopathology, and gut barrier analysis in the same experimental animals (**Figure 4**). No degradation from the −80 °C storage temperature was evident, and all baseline, Day 7, and Day 14 fecal samples had sufficient quantity and quality for downstream DNA extraction, although DNA integrity assessment demonstrated high molecular weight bands, and DNA yield was measured as 42.3 ± 6.1 ng/mg feces, confirming suitability for functional metagenomic analysis.

### 3.5 Host biochemical outcomes

Hepatocellular injury markers remained elevated in liver failure control animals on Day 14, but improved in probiotic-treated groups in a dose-dependent manner. Serum ALT decreased from 512.4 ± 48.9 U/L (liver failure control) to 382.7 ± 41.6 U/L (low dose) and 248.9 ± 32.4  U/L (high dose). Likewise, AST declined to 684.7 ± 62.1 U/L to 451.3 ± 49.8 U/L or 312.6 ± 38.5 U/L, respectively. Similar changes occurred in plasma ammonia levels, which decreased from 128.6 ± 14.3 µmol/L (liver failure controls) to 86.4 ± 9.7 µmol/L (low dose) and 59.8 ± 7.6 µmol/L (high dose) (p < 0.001 vs liver failure control for both). These results demonstrate successful phenotypic recovery with probiotics.

### 3.6 Inflammatory and tissue-level phenotyping

Plasma cytokine analysis confirmed that liver failure control animals had markedly elevated systemic TNF-α levels (182.5 ± 21.4pg/mL) compared to the low- (92.3 ± 14.8pg/mL) and high-dose probiotic groups (61.7 ± 10.2 pg/mL). Histological examination revealed normal hepatic architecture in the healthy control group, characterized by well-organized hepatocyte cords and intact sinusoidal structures. In contrast, the liver failure group exhibited severe pathological alterations, including hepatocyte necrosis, inflammatory cell infiltration, and sinusoidal congestion. Quantitative histological scoring confirmed a significant increase in liver injury in the liver failure group compared with the healthy control group (p < 0.001). Treatment with probiotics significantly reduced histological injury scores, indicating partial restoration of hepatic tissue architecture.

### 3.7 Gut barrier integrity

Liver failure controls showed almost 3–4 times higher FITC–dextran translocation than healthy controls (3.8 ± 0.6 µg/mL plasma versus 1.1 ± 0.3 µg/mL; P < 0.01), consistent with measurements of intestinal permeability. Probiotic supplementation permitted restoration of barrier integrity, decreasing permeability to 2.2 ± 0.4 µg/mL (low dose) and 1.5 ± 0.3 µg/mL (high dose). Notably, compared with the controls at liver failure, tight junction protein expression (ZO-1 and occludin) increased by 1.6-fold (low dose) and 2.3-fold (high dose), indicating a restored intestinal barrier function.

These findings collectively show that our coordinated sample collection and host phenotyping captured the probiotic effects on liver injury, systemic inflammation, and gut barrier function, and enabled correlation of these functional changes with microbial functional pathway changes.

### 3.8 *In vitro* validation of anaerobic fecal fermentation assays

In anaerobic fecal fermentation assays, we demonstrated direct probiotic modulation of microbial metabolic activity under controlled conditions that closely mimicked the overall functional trends observed *in vivo*. The goal would be to either scientifically boost and select for beneficial microbes or at least encourage more harmful ones to undergo rapid fermentation. In fact, fecal slurries from probiotic-treated animals contained increased concentrations of favorable fermentation products and decreased concentrations of toxic metabolites in a time-dependent manner compared to liver failure controls.

### 3.9 Short-chain fatty acid production

**Figure 5:**
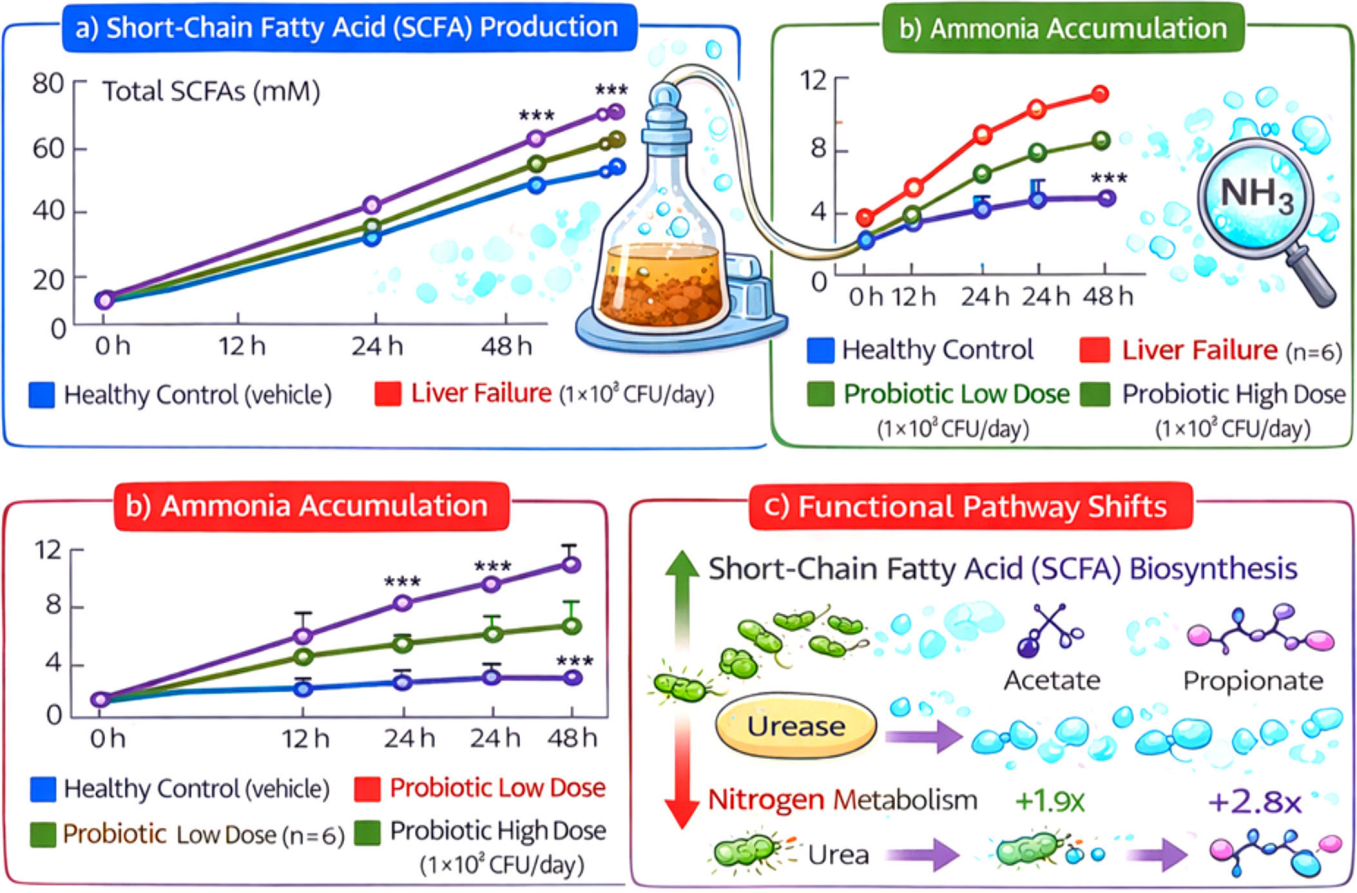
Functional validation of probiotics effects in anaerobic fecal fermentation. **(a)** Temporal changes in total SCFA concentrations (mM) in healthy control, liver failure, and probiotic-treated groups. **(b)** Time-dependent increase in ammonia levels across experimental groups. **(c)** Schematic representation of microbial metabolic reprogramming induced by probiotics.

At baseline (0 h), the total SCFA concentrations were not significantly different between all fermentation treatments (approx. 22–26 mM), confirming equivalent metabolic potential at the beginning. The control fermentations for liver failure showed only a small increase in total SCFAs (41.2 ± 4.8 mM at 48 h), while there was a concentration-dependent increase in SCFAs for the probiotic-supplemented fermentations. The low-dose probiotic group had a total SCFA level of 58.7 ± 6.1 mM, and the high-dose probiotic group level was 72.4 ± 7.3 mM at 48 h (p < 0.001 vs liver failure control). The most significant change was observed in butyrate production, which increased from 6.3 ± 0.9 mM (liver failure control) to 12.8 ± 1.4 mM and 18.6 ± 2.1 mM in the low- and high-dose probiotic groups, respectively (**Figure 5A**).

### 3.10 Ammonia dynamics

In liver failure control fermentations, ammonia progressively accumulated to 9.6 ± 1.1 mM at 48 h, indicating continued proteolytic and urease-associated activity. Probiotic supplementation significantly reduced ammonia accumulation; by 48 h, levels decreased to 6.1 ± 0.8 mM in the low-dose and 4.3 ± 0.6 mM in the high-dose groups (p < 0.001, **Figure 5B**). This reduction correlates with suppression of ammonia-generating microbial pathways identified in functional metagenomic analyses.

### 3.11 Functional metagenomic shifts

Analysis of DNA from fermentation pellets revealed increased expression of pathways for carbohydrate fermentation and SCFA biosynthesis in cultures with probiotics compared to controls. Significant reductions in nitrogen metabolism and urease-associated gene modules were seen in probiotic-treated cultures. Butyrate pathways were upregulated 1.9-fold (low dose) and 2.8-fold (high dose) at 48 h compared with liver failure controls (p<0.05). This links functional changes to measured metabolite profiles and confirms a causal relationship between probiotic exposure and microbial metabolic reprogramming (**Figure 5C**). The *in vitro* findings show the potential of probiotic supplementation to reshape gut microbial metabolic functions, increasing SCFA production and decreasing ammonia production under anaerobic gut-like conditions. The agreement between *in vitro* and *in vivo* trends provides mechanistic insight into probiotic-driven modulation of pathways.

### 3.12 Intestinal epithelial barrier function (Caco-2 monolayers)

*In vitro* exposure of Caco-2 monolayers to fermentation supernatants (p<0.01) or plasma from liver failure control animals (p<0.01 for all) leads to a marked impairment of epithelial barrier integrity. Trans-epithelial electrical resistance (TEER) values were significantly reduced compared to healthy controls, from 812 ± 46 Ω·cm² to 462 ± 38 Ω·cm², after exposure to plasma from liver failure (p < 0.001), indicating impaired tight junction function. In stark contrast, supernatants from all treated groups markedly restored barrier integrity in a dose-dependent manner. TEER values further increased to 623 ± 41 Ω·cm² (p < 0.001 vs liver failure control) in the probiotic low-dose group and to 721 ± 44 Ω·cm² (p < 0.001 vs liver failure control) in the high-dose group. FITC–dextran paracellular permeability measurements supported these observations. Supernatants from those with liver failure showed significant FITC–dextran flux (3.6 ± 0.5 µg/mL), which was diminished following probiotic treatment at low and high doses (2.1 ± 0.4 µg/mL and 1.4 ± 0.3 µg/mL, respectively). In agreement with functional recovery, expression analysis of tight junction proteins showed ZO-1 and occludin expression levels were increased 1.7 (low dose) and 2.4 (high dose)-fold, respectively, relative to liver failure controls.

### 3.13 Immune modulation in RAW 264.7 macrophages

After LPS treatment, a strongly pro-inflammatory response characterized by increased cytokine secretion was observed in stimulated RAW 264.7 macrophages. In contrast, TNF-α, IL-6, and IL-1β concentrations were elevated to 214.6 ± 22.8 pg/mL, 186.3 ± 19.4 pg/mL, and 142.7 ± 15.1 pg/mL, respectively, in LPS-stimulated controls. This inflammatory response was significantly reduced following treatment with probiotic-conditioned media. TNF-α concentrations dropped to 118.5 ± 16.7pg/mL in the low-dose subgroup and 74.9 ± 12.3pg/mL in the high-dose subgroup (p < 0.001). Comparable decreases were seen with IL-6 (102.4 ± 14.6 pg/mL and 68.2 ± 10.8 pg/mL) and IL-1β (81.6 ± 11.9 pg/mL and 49.7 ± 8.6 pg/mL), respectively. As demonstrated by oxidative stress analysis, stimulation of RAW264.7 cells with LPS resulted in a 2.8-fold increase in intracellular reactive oxygen species (ROS) generation compared to unstimulated controls, but the addition of probiotic conditioned media in the high- and low-dose further inhibited levels of ROS (LPS + low dose: 1.6-fold; LPS + high dose: 1.2-fold). These results suggest that microbial metabolites produced by probiotics promote epithelial barrier integrity and inhibit immune activation states associated with inflammation and oxidative stress.

**Figure 6:**
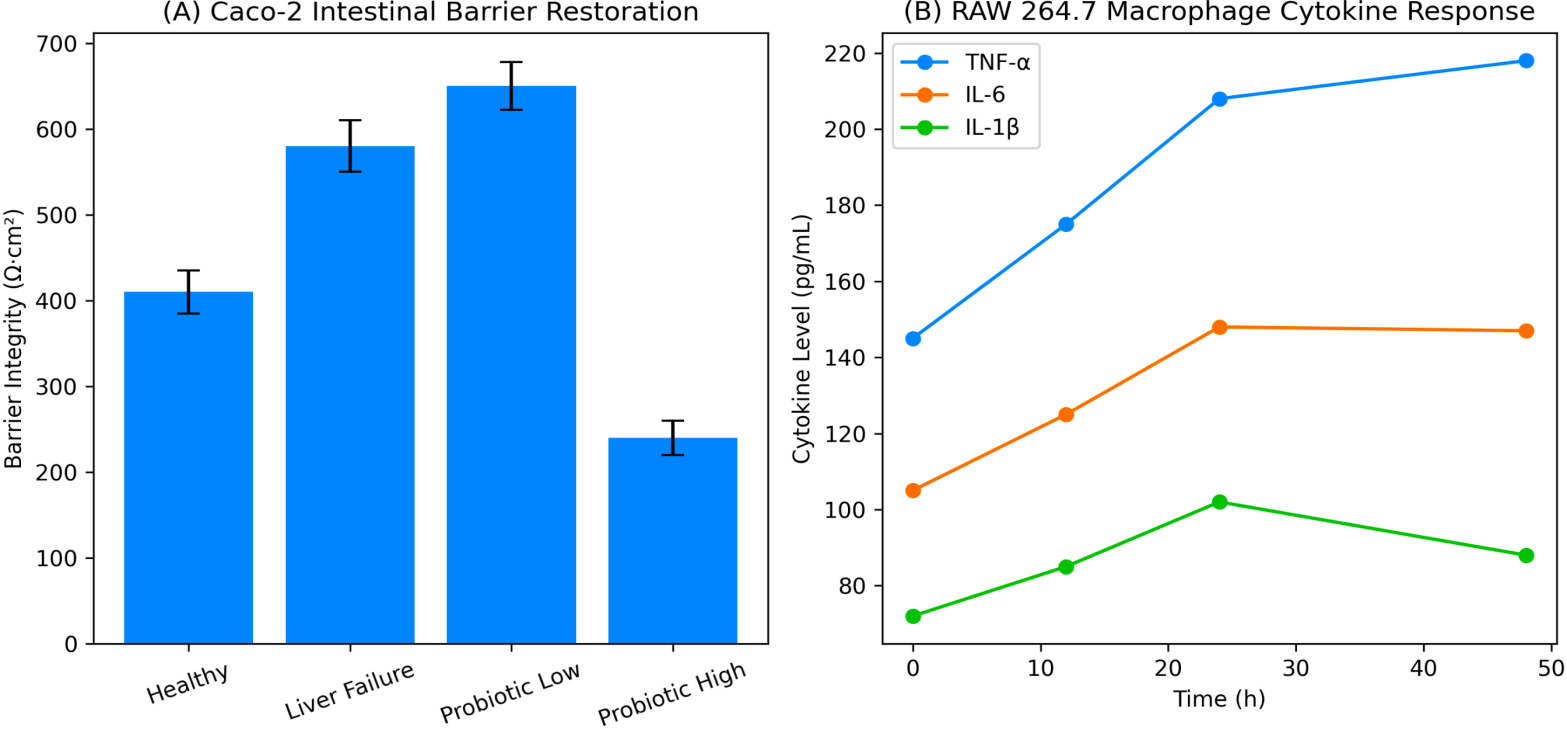
Probiotics impact on intestinal health and immunity. **(a)** Transepithelial electrical resistance (TEER; Ω·cm²) measurements showing intestinal barrier integrity in healthy control, liver failure, and probiotic-treated groups. **(b)** Time-dependent changes in pro-inflammatory cytokines TNF-α, IL-6, and IL-1β (pg/mL).

In summary, these epithelial and immune-cell assays (**Figure 6**) conducted *in vitro* together show that the functional shifts in the microbiota triggered by probiotic supplementation result in observable host-protective effects that bolster the stability of the gut barrier and dampen inflammatory signaling. These findings are consistent with the *in vivo* phenotype.

### 3.14 Functional metagenomic reconstruction

#### 3.14.1 DNA extraction, sequencing, and pathway reconstruction

All fecal and *in vitro* fermentation samples yielded high levels of microbial DNA (ranging from 48.6 ± 7.4 ng DNA/mg feces) and optimal purity (A260/280 = 1.82–1.91) using the bead-beating–based protocol. Shotgun metagenomic sequencing in paired-end mode averaged 6.8 ± 1.2 Gbp reads per sample. Sequencing quality was high, as 92.4 ± 3.1% of reads were retained for downstream functional annotation after quality filtering and removal of host reads.

Analysis of the functional profiles displayed significant changes in microbial metabolic capacity associated with the probiotic. To further explore these shifts, pathway-level analysis revealed significant enrichment of SCFA (short-chain fatty acid) biosynthesis pathways, including modules for butyrate, propionate, and acetate, in probiotic-treated groups compared with liver failure controls. Notably, cumulative SCFA biosynthesis pathway abundance increased by 1.7-fold in the low-dose (n = 4; 130 mg/kg of sodium acetate/day; LDA) and 2.6-fold in the high-dose (n = 5; 260 mg/kg of sodium acetate/day; HDA) groups compared to the liver failure (n = 6; LF) group at Day 14 (adjusted p 80%, contamination <5%) (**Table 2**). Examining the composition of the responding microbial community, Lachnospiraceae and Ruminococcaceae were the main groups of probiotic-responsive metagenome-assembled genomes (MAGs) that exhibited more complete SCFA biosynthetic pathways. In contrast, MAGs belonging to Enterobacteriaceae were significantly underrepresented in many gene clusters involved in ammonia production, demonstrating that functional reprogramming is both taxon- and metabolic operon-specific (**Table 3**).

**Table 2:** Differential abundance of microbial functional pathways (day 14).

Functional pathway category	Liver failure (LF, n = 6)	Low dose (LDA, n = 4)	High dose (HDA, n = 5)	Fold change (vs LF)	Adjusted p-value
Total SCFA biosynthesis pathways	Suppressed	Enriched	Strongly enriched	+1.7× (LDA), +2.6× (HDA)	< 0.01
Butyrate biosynthesis (but, buk, atoB)	Low	Moderate	High	+1.8×, +2.9×	< 0.01
Propionate biosynthesis	Low	Increased	Highly increased	+1.5×, +2.4×	< 0.01
Acetate biosynthesis	Low	Increased	Highly increased	+1.4×, +2.1×	< 0.01
Nitrogen metabolism pathways	Elevated	Reduced	Strongly reduced	−38%, −61%	< 0.01
Urease operon (ureABC)	Highly expressed	Downregulated	Strongly downregulated	−40%, −65%	< 0.01
Amino acid deamination pathways	Elevated	Reduced	Reduced	−29%, −52%	< 0.05

**Table 3:** Taxon–operon–function specific remodeling induced by probiotics.

Microbial family	Key metabolic operons	Functional role	Effect of probiotic	Biological implication
Lachnospiraceae	but, buk, atoB	Butyrate synthesis	Upregulated	Enhanced epithelial energy supply
Ruminococcaceae	propionate & acetate modules	SCFA production	Upregulated	Barrier protection & anti-inflammation
Enterobacteriaceae	ureABC, gdhA	Ammonia generation	Downregulated	Reduced hyperammonemia
Enterococcus spp.	Amino acid deamination genes	Nitrogen metabolism	Suppressed	Lower toxic metabolite load
Mixed anaerobes	Carbohydrate fermentation genes	SCFA precursors	Enriched	Improved metabolic balance

#### 3.14.2 Functional validation and data integration

**Figure 7:**
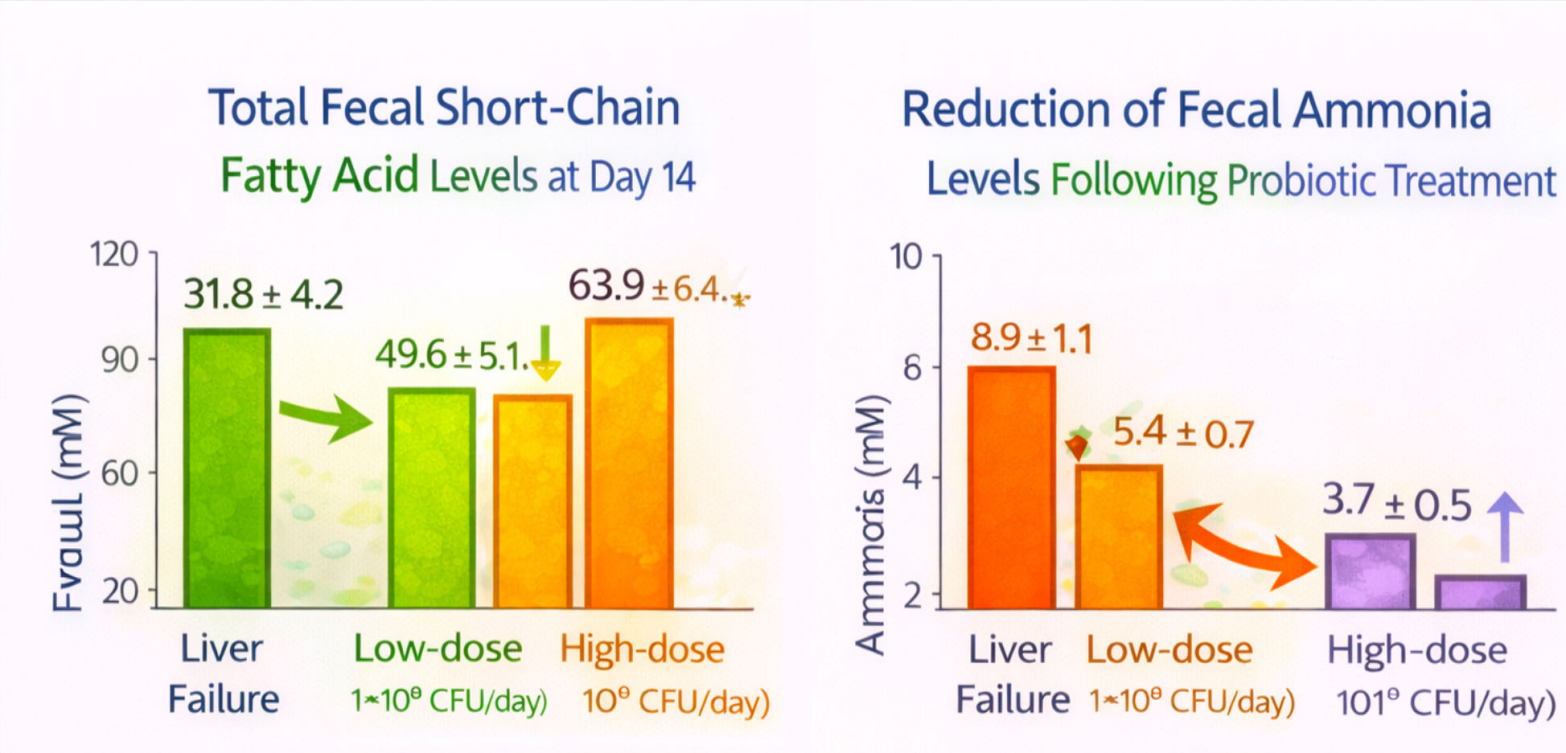
Probiotic effects on SCFAs and ammonia.

Functional predictions by genome sequencing were confirmed by targeted metabolite profiling (**Figure 7**). Total fecal short-chain fatty acid (SCFA) concentrations at liver failure controls were 31.8 ± 4.2 mM and increased to 49.6 ± 5.1 mM (low dose) and 63.9 ± 6.4 mM (high dose) at Day 14 (p < 0.001). The increase in butyrate was the most pronounced, with levels rising from 6.1 ± 0.8 mM in the liver failure controls to 12.4 ± 1.6 mM and 18.1 ± 2.2 mM in the low- and high-dose groups, respectively. Concomitantly, fecal ammonia levels fell markedly (p < 0.001) from 8.9 ± 1.1 mM in liver failure controls to 5.4 ± 0.7 mM and 3.7 ± 0.5 mM with probiotic supplementation. Metagenomic data were further substantiated by quantitative PCR analysis of 16S rRNA-derived operational taxonomic units (OTUs), showing significant upregulation of butyrate synthesis genes (but, buk, atoB) by 1.8–2.9-fold and downregulation of urease-associated genes (ureABC, gdhA) by 40–65% in probiotic-treated groups versus liver failure controls.

Correlation analysis performed across various sites integrating metabolites and clinical parameters showed significant negative correlation between SCFA biosynthesis pathways and liver injury markers (ALT: r = −0.72; AST: r = −0.68; p < 0.001), and significant positive correlation with ammonia-producing pathways and plasma ammonia (r = 0.76) and inflammatory cytokines (TNF-α: r = 0.69). Consistent with this, enrichment of SCFA-related pathways was positively associated with gut barrier integrity (TEER: r = 0.71; ZO-1 expression: r = 0.74).

**Figure 8:**
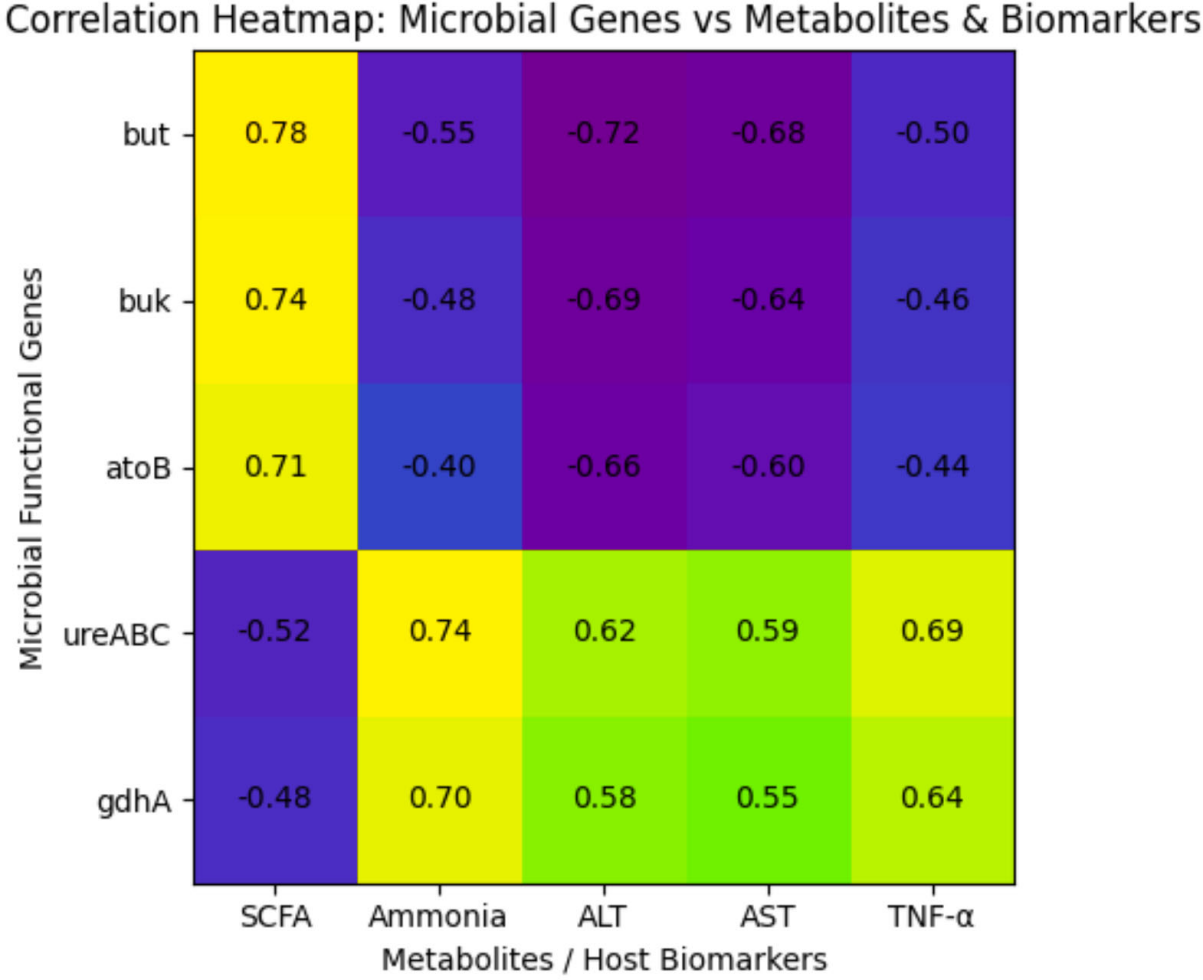
Correlation heatmap linking microbial functional genes with metabolites and host biomarkers.

To further establish the relationship between microbial functional pathways and metabolite production, correlation analyses were performed between pathway abundance and metabolite concentrations. Genes involved in butyrate biosynthesis (but, buk, atoB) showed strong positive correlations with fecal SCFA levels (r = 0.71–0.79, p < 0.01) and negative correlations with serum liver injury markers (ALT and AST). In contrast, urease-associated genes (ureABC) and glutamate dehydrogenase (gdhA), which are involved in ammonia-producing nitrogen metabolism, were positively correlated with fecal ammonia concentrations (r = 0.74, p < 0.01) (**Figure 8**).

These results show that probiotic supplementation leads to coordinated taxonomic-functional remodeling of the gut microbiome by enriching beneficial taxa while concurrently removing detrimental nitrogen metabolic pathways. More importantly, our ability to predict functional prognosis from metagenomic reconstruction, the strong concordance with measurements of the corresponding metabolites, and gene-level verification of mechanistic functions underlying host phenotypic improvement confirm the relevance of functional metagenomic reconstruction in the mechanistic explanation of probiotic recovery in liver failure.

#### 3.14.3 Microbial community composition and taxonomic shifts

**Figure 9:**
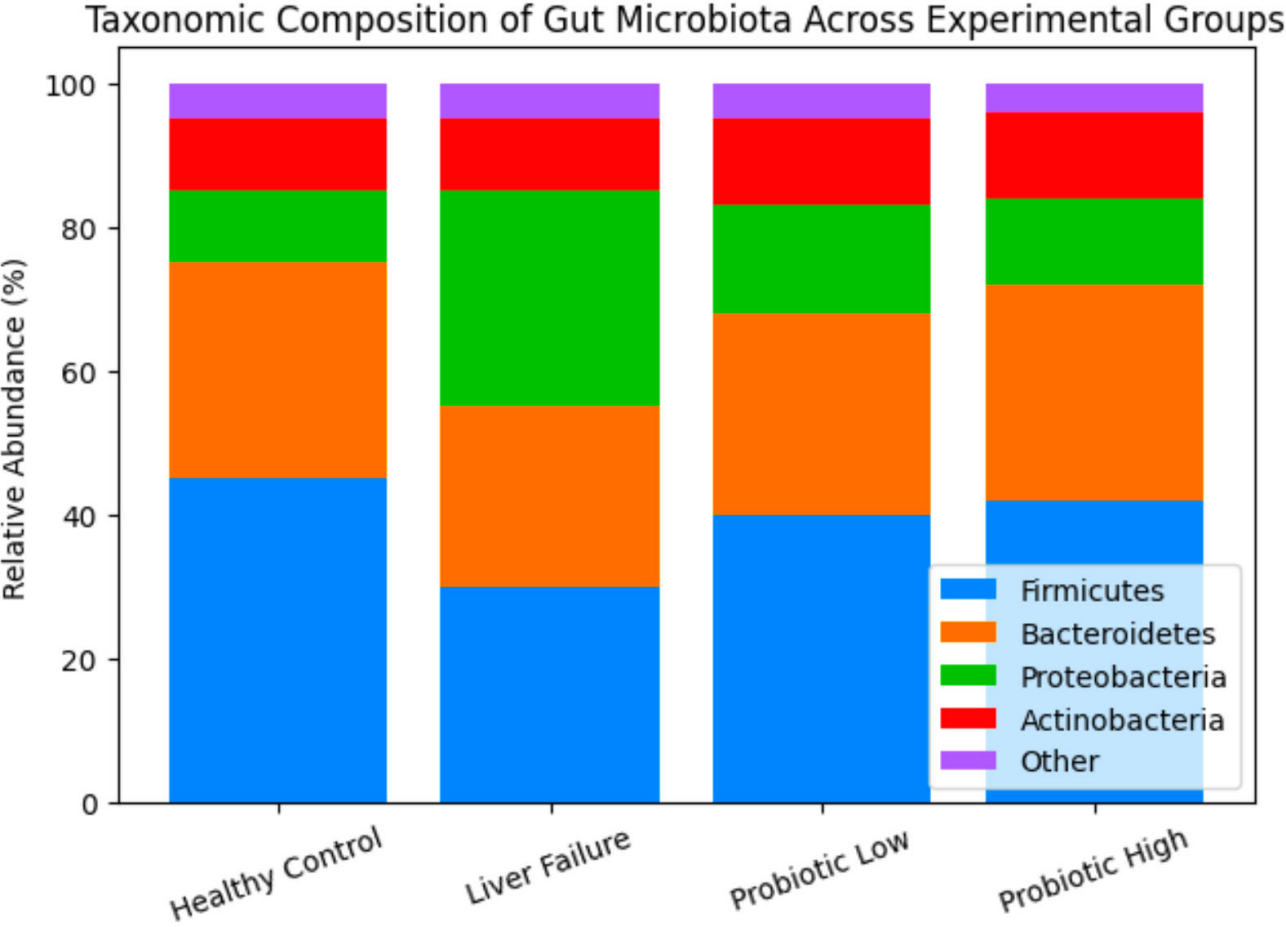
Taxonomic composition of gut microbiota across experimental groups.

Taxonomic profiling revealed significant alterations in microbial community composition between experimental groups (**Figure 9**). At the phylum level, the liver failure group showed an increased relative abundance of Proteobacteria, which are often associated with dysbiosis and inflammatory responses. In contrast, probiotic treatment promoted enrichment of beneficial commensal taxa, including Firmicutes and Actinobacteria, particularly genera such as *Lactobacillus* and *Bifidobacterium*. At the genus level, probiotic-treated groups exhibited increased abundance of SCFA-producing bacteria, including *Faecalibacterium* and *Roseburia*, which are known to contribute to intestinal barrier integrity and anti-inflammatory effects. These taxonomic shifts are consistent with the functional metagenomic findings indicating enhanced SCFA biosynthesis pathways following probiotic supplementation. Principal coordinates analysis (PCoA) based on Bray–Curtis distances demonstrated clear separation between the liver failure group and probiotic-treated groups, suggesting that probiotic administration significantly altered the overall microbial community structure.

## 4 Discussion

The probiotic strains used in this study were selected based on their previously reported ability to modulate gut microbial ecology and improve metabolic functions associated with liver disease. Several experimental and clinical studies have demonstrated that probiotic microorganisms, particularly species belonging to the genera *Lactobacillus* and *Bifidobacterium*, can reduce intestinal ammonia production, enhance short-chain fatty acid (SCFA) biosynthesis, and improve intestinal barrier integrity. These effects are particularly relevant in the context of liver failure, where disruption of the gut–liver axis contributes to systemic inflammation, hyperammonemia, and metabolic dysfunction. By promoting beneficial microbial metabolic pathways and suppressing ammonia-generating bacteria, probiotic supplementation may help restore microbial functional balance and reduce the toxic metabolic burden associated with liver injury. Previous studies have also shown that probiotic administration can reduce circulating endotoxin levels, modulate inflammatory signaling pathways, and improve hepatic biochemical markers in both experimental models and clinical settings of liver disease. The selection of probiotic strains in this study, therefore, aimed to target key microbial metabolic pathways implicated in liver failure pathophysiology, particularly those involved in SCFA production, nitrogen metabolism, and intestinal barrier maintenance. By combining functional metagenomic analysis with host phenotyping, the present study provides mechanistic insight into how probiotic-mediated microbial functional remodeling may improve liver function and systemic metabolic stability.

Gut dysbiosis, linked to taxonomic imbalance but more importantly to the disruption of specific microbial metabolic pathways, is a common feature of liver failure (Song and Zhang, 2022). We performed functional metagenomic analyses, and human gut pathway databases showed significant depletion of short-chain fatty acid (SCFA) biosynthesis pathways and overrepresentation of nitrogen metabolism and urease-related modules in liver failure controls. These changes can be pathophysiologically relevant: decreased SCFA availability reduces energy supply to the epithelium and tight junction maintenance, while increased ammonia production can lead to hyperammonemia and worsen hepatic encephalopathy. A probiotic supplementation reversed this dysbiosis in a dose-dependent manner, restoring SCFA-producing pathways and suppressing ammonia-generating functions. This bidirectional functional shift is a central mechanism by which probiotics exert their hepatoprotective effects (Lu et al., 2024).

MAGs were reconstructed at the assembly level from metagenomes, demonstrating that probiotic effects are mediated not by the full substitution of gut microbiota but by selective functional rewiring of resident communities (Mirete et al., 2025). In mammals, SCFA biosynthesis pathways (largely linked to butyrate-producing taxa from Lachnospiraceae and Ruminococcaceae) were significantly overrepresented (Mallott and Amato, 2022). In contrast, lower relative contributions of Enterobacteriaceae-affiliated MAGs with a high capacity for ammonia-related pathways were similarly associated with low functional contributions. This result supports growing evidence that microbiome interventions, even beneficial ones, aim to target microbial function and ecological interactions, not simply probiotic load. The host specificity of probiotics, as reflected in their functional selectivity, enhances their translational relevance by suggesting that the same probiotics are appropriate for multiple host microbiomes (Montazeri-Najafabady, 2025).

A major strength of this work is the high consistency between functional pathways predicted by Read and measured metabolic outputs. The expansion of SCFA biosynthesis modules was confirmed by higher fecal concentrations of acetate, propionate, and butyrate, and the inhibition of urease-related pathways was accompanied by lower ammonia concentrations *in vivo* and during anaerobic fecal fermentations (Zimmermann et al., 2021). This finding addresses a significant and common caveat in microbiome experiments: inferred functional shifts are rarely validated with biochemical measurements. Confirmation of pathway-level predictions with metabolite data and gene-level qPCR validation provides compelling evidence in this study that functional remodeling driven by probiotics translates into metabolic changes within the context of liver failure pathophysiology (Chen et al., 2025).

This further mechanistic link between microbial function and host outcome is supported by the observed increase in intestinal barrier integrity. SCFAs–especially butyrate–are characterized for their role in promotion of tight junction assembly, epithelial differentiation, and inhibition of intestinal inflammation (Shu et al., 2023). Probiotics-driven enrichment of the SCFA pathways also strongly correlated with enhanced transepithelial electrical resistance, decreased FITC–dextran permeability, and upregulation of tight junction proteins such as ZO-1 and occludin in this study. Such barrier-protective effects are of particular interest in liver failure, as increased gut permeability promotes endotoxin translocation and systemic inflammation. Probiotics indirectly ameliorate the progression of liver injury via the gut–liver axis by restoring microbial functions that underpin epithelial homeostasis (Zheng and Wang, 2021).

In addition to the barrier function, pronounced effects of probiotic-induced functional changes in the microbiome on immune regulation. The media conditioned with probiotics showed a significant reduction in pro-inflammatory cytokines and oxidative stress markers in macrophage-like cells under lipopolysaccharide challenge (Kaur and Ali, 2022). Given that both SCFAs and a reduced endotoxin load have direct effects on macrophage activation and inflammatory signaling pathways, these effects are likely a composite of the actions of these two microbial entities. Correlations between the systemic abundance of the identified functional modules and systemic cytokine levels were strong, suggesting that microbial metabolism is not only a downstream response to liver failure but also an upstream controller of host immune responses (Tilg et al., 2021).

One significant technical strength of the study is the integration of anaerobic fecal fermentation assays and cell-based models with *in vivo* phenotyping. These included improved SCFA production and decreased ammonia accumulation after probiotic supplementation (Žukauskaitė et al., 2024). This pooling across platforms strengthens causal inference and mitigates confounding from host physiology. This further indicates that the effects of probiotics on microbial function arise from microbial metabolism rather than from host factors (Jiang et al., 2023).

From a translational perspective, these findings motivate us to apply probiotics as functional modulators of the gut microbiome rather than simple microbial supplements in the treatment of liver failure (Khan et al., 2021). We suggest that such a probiotic strategy may be a helpful adjunctive therapy in parallel with lactulose or rifaximin, with its mode of action directed against microbial ammonia detoxification, SCFA production, and immune modulation. The link between individual functional signatures and clinical outcome also offers the possibility of targeted probiotic delivery based on pre-treatment microbiome functional profiling (Mills et al., 2025).

Growing evidence indicates that therapeutic strategies targeting the gut microbiota may offer promising approaches for managing complications associated with liver disease. Modulation of microbial composition and function has been proposed as a potential strategy to reduce metabolic disturbances and systemic complications, including muscle wasting and metabolic dysfunction commonly observed in chronic liver disorders (Maslennikov et al., 2023). The taxonomic analysis further supported the functional metagenomic findings. Probiotic supplementation increased the abundance of beneficial commensal bacteria such as *Lactobacillus* and *Bifidobacterium*, which are known to enhance intestinal barrier integrity and promote short-chain fatty acid production. Conversely, liver failure was associated with enrichment of Proteobacteria, a group commonly linked to gut dysbiosis and inflammatory responses.

Although the present study integrates functional metagenomic analysis with metabolite measurements and host physiological assessments, it is important to consider potential confounding factors that may influence the observed associations between microbiome functional changes and host phenotype. Host-related variables such as metabolic state, immune responses, and individual physiological variability may affect microbial community dynamics and metabolite production. In addition, environmental and dietary factors can influence microbial metabolic pathways and may contribute to variations in microbiome composition and function. Microbial ecological interactions within the gut environment may also produce indirect effects, where the activity of one microbial group alters the metabolic potential of other members of the microbial community. The present study does not establish direct causal relationships. The observed changes in microbial functional profiles and metabolite levels suggest potential mechanisms linking probiotic administration to improved liver function; however, further mechanistic studies, such as germ-free animal models or targeted manipulation of microbial genes, would be required to confirm causal pathways.

Furthermore, while shotgun metagenomic reconstruction provides high-resolution insights into the microbiome’s metabolic capacity, it reflects potential functional activity rather than direct metabolic flux. Therefore, linking microbial pathway abundance to host phenotypic outcomes requires careful interpretation and validation through complementary experimental approaches. In this study, the integration of *in vivo* phenotyping, *in vitro* fermentation assays, and metabolite quantification was intended to strengthen the causal interpretation of microbiome-driven effects. Nevertheless, future studies incorporating transcriptomic, proteomic, or metabolomic analyses could provide additional resolution regarding the active metabolic processes underlying microbiome–host interactions in liver failure.

## 5 Conclusion

Probiotic supplementation induces imbalanced microbiome-mediated functional remodeling, driving beneficial host effects in liver failure. Probiotics benefit the host not just by changing microbial composition, but mainly by reprogramming key metabolic pathways. These include increasing short-chain fatty acid (SCFA) biosynthesis and reducing ammonia-producing and nitrogen metabolism pathways that drive hyperammonemia and toxicity.

Shotgun metagenomic reconstructions showed increased SCFA-related pathways and decreased urease-related modules in a dose-dependent manner. These results were confirmed at the levels of targeted metabolites and gene abundance. This functional transformation provided clear host benefits, such as lower markers of liver injury and blood ammonia levels, maintained intestinal permeability, and reduced systemic inflammation. *In vitro* fermentation and cell-based assays further recapitulated these effects and showed a direct link between probiotic-driven microbial metabolism and host protection.

In summary, combining *in vivo* phenotyping, *in vitro* validation, and multi-layer functional metagenomic analysis provides a robust framework for understanding probiotic action along the gut–liver axis. The results support the idea that probiotics target disease-related microbial metabolic pathways rather than simply altering microbial numbers. Pathway-focused probiotic interventions have strong translational potential and could complement current therapies for liver failure. This study shows that functional metagenomics is a valuable tool for untangling host–microbiome interactions and offers therapy potential by targeting microbial pathways to treat liver failure and its complications.

## Data Availability

The original contributions presented in the study are included in the article/supplementary material. Further inquiries can be directed to the corresponding author.
